# Incidence of Cervical, Ovarian and Uterine Cancer in Eritrea: Data from the National Health Laboratory, 2011-2017

**DOI:** 10.1038/s41598-020-66096-5

**Published:** 2020-06-04

**Authors:** Lidia Biniam Medhin, Lia Alem Tekle, Oliver Okoth Achila, Salih Said

**Affiliations:** 1 Pathology, National Health Laboratory, Asmara, Eritrea; 2 Microbiology, National Health Laboratory, Asmara, Eritrea; 3 Biochemistry, Orotta School of Medicine and Health Sciences, Asmara, Eritrea

**Keywords:** Cancer epidemiology, Gynaecological cancer

## Abstract

The main objective of this study was to investigate the incidence of cervical (C53), ovarian (C56) and uterine (C54–55) cancers in pathology department of the National Health Laboratory of Eritrea between 2011 and 2017. All tumour positive cases from cervix, ovary and uterus diagnosed between 2011 and 2017 were analyzed, based on the data from the pathology department available in National Health Laboratory. We summarized the results by using crude incidence rates (CIR) and age-standardized rates (ASRs). Annual percentage changes (APCs) for each site were calculated and compared according to ten-year age difference and year of occurrence. Between 2011 and 2017, 883 cases of cervical, ovarian and uterine tumours were reported in Eritrea. Malignant and benign tumours/entities comprising 269 and 614, respectively. The ASR for malignant tumours was highest in women aged between 60–69 (6.84 per 100 000). Total ASR for specific gynecological cancers (cervical, ovarian, uterine) was 19.32 per 100 000 females. The ASR for cervical cancer over the study period was 8.7 per 100 000. The ASR for ovarian and uterine cancers were 6.75 per 100 000 and 5.14 per 100 000, respectively. Over the study period, the incidence of these cancers was largely stable with no significant change in incidence rates recorded. In sum, the ASR for cervical cancer is relatively low compared to the rates reported in the region. Further, the ASR for ovarian and uterine cancers is nearly similar to the rates observed in this region. The study also provides ample evidence on the need for research targeted at uncovering the true burden of gynecological cancers in Eritrea. Potential solutions will require the establishment of high-quality population-based cancer registries (PBCRs) and long-term commitment to improvements in research platforms, training, screening, diagnosis, and the overall management of cancers in the country.

## Introduction

Gynecological cancers are common cancer with high mortality worldwide. Cervical cancer is the cancer that forms on the tissue of the cervix which is the organ connecting the Uterus and Vagina. Uterine cancer forms in the tissue of the uterus and have two types, endometrial cancer (cancer that begins in cells lining of the uterus) and Uterine Sarcoma (a rare type that begins in the muscle or other tissues in the uterus) while ovarian cancer forms in the tissues of the ovary. Cancers of the cervix (C53), uterus (C54–55) and ovaries (C56) contribute significantly to female morbidity and mortality worldwide^1^. With an estimated 119 284 cases and 81 687 deaths in 2018 in Africa, cervical cancer ranks 2^nd^ and 1^st^ for both incidence and mortality^2^. High-risk regions (with estimated age-standardized incidence rates ASIR) of >30 per 100,000 women) include eastern Africa (42.7), and southern and middle Africa (31.5 and 30.6 respectively)^2^. According to the International Agency for Research on Cancer (IARC) Global Burden of Cancer (GLOBOCAN) database, the incidence of ovarian cancer was 21 925 cases in 2018 making it the 12^th^ cause of death from cancer in Africa^3^. The same report indicates that Corpus uteri is ranked 20^th^ in terms of incidence in Africa^4^. In general, prevalence of ovarian and uterine cancers is higher in high income countries, particularly northern Europe and North America, and lower in low-and medium-income countries (LMIC) in Asian and sub-Saharan Africa (SSA)^4^. In contrast, incidence rates are much higher for cervical cancer in LMIC, where 80% of all cases occur^5^. With the on-going cancer transitions in Sub-Saharan Africa (SSA), it is estimated that, by 2030, cancer will become the cause of over 13 million deaths a year^3^.

The mortality profile among women for these cancers is more heterogeneous and varies substantially, across and within countries depending on the Human Development Index (HDI) and accompanying social and life style factors. For instance, cervical cancer ranks 2^nd^ in mortality behind breast cancer in low-HDI settings; however, it is the leading cause of cancer death in 42 countries – mostly in SSA and South-Eastern Asia^6^. In addition, some projections indicate that cervical cancer mortality is expected to increase in LMIC by 42% to 442 926 deaths by 2030^5^. Similarly, ovarian cancer has a very high case fatality rate that results from diagnosis of advanced (Stage III and IV)^7^. Studies indicate that the case fatality ratio for ovarian cancer is generally greater than 50% and over 70% in East Africa (EA)^6^. Moreover, women from rural areas account for a disproportionate fraction of these cases^8^. Overall, cervical cancer is a greater source of morbidity and mortality than uterine and ovarian cancers combined.

Despite the high mortality associated with gynaecological cancers in SSA; the true burden is largely unknown. Predictably, little data exist on the incidence or mortality rates of gynaecological malignancies in SSA^1,3^. According to the most recent data from IARC, even low – quality population-based cancer registry (PBCR) data, the basis for planning and implementing evidence-based cancer intervention programs, are not available (Eritrea included)^4^. In addition, data from existing registries in SSA is largely questionable - many of these registries cover subnational areas, such as selected urban areas and critical information on the patients is lacking^2,9,10^. Population denominators and the accuracy of data from some reports have also been questioned. As a consequence, past IARC country specific estimates for countries like Eritrea were modelled from cancer registry data in neighbouring countries^2^. Therefore, the main aim of this study was to provide data on the incidence of specific gynaecological cancers (Ovarian, uterine and cervical) in Eritrea. Cancer incidence of these specific sites was chosen as they are the top incident gynaecological cancers in the world along with breast cancer, and incidence of breast cancer in Eritrea from 2011–2017 has been studied in previous research. To this end, data abstracted from the pathology department of National Health Laboratory (NHL) in Asmara, Eritrea, was utilised.

## Results

**Figure 1:**
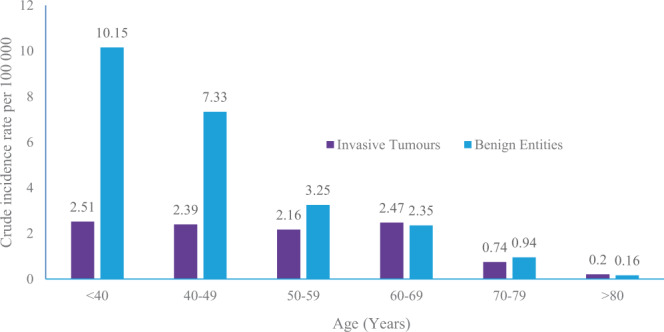
Crude incidence rates of gynecological tumour cases (2011–2017) per age group.

Between 2011 and 2017, 883 cases of cervical, ovarian and uterine tumours were recorded. The proportion of benign and malignant entities was 614 and 269 respectively. Age-related difference in the magnitude of CIR was observed with women less than 40 years presenting with the highest CIR (10.15 per 100 000) of benign tumours/entities. A significant decrease in CIR of benign tumours was recorded for subsequent age groupings with lowest rates reported in individuals >80 years of age (0.16 per 100 000). In contrast, lowest CIR for malignant tumours was observed in individuals >80 years (0.16 per 100 000). Highest CIR for malignant tumours was observed in individuals between 60–69 years of age (2.47 per 100 000) (Fig. 1).

### Incidence rates of cervical, ovarian and uterine cancer between 2011–2017

Between 2011 and 2017, the pooled ASR for specific gynecological cancers (cervical, ovarian, uterine) was 19.32 per 100 000 females. The ASR for cervical, uterine and ovarian cancers were 8.7 per 100 000; 5.14 per 100 000 and 6.75 per 100 000, respectively. Table 1 shows CIR, ASR and APC for all cases. Estimated APC over the period for cervical cancers was – 0.43 (95% CI, − 18.3–23.5, p > 0.8). The APC for uterine and ovarian cancers were 5.64 (95% CI, − 13.8–29.4) and –11.26 (95% CI, −28.6–10.3) see Table 1.

**Table 1:** ASR per 100,000 by year for each site.

Year of diagnosis	Cervix		Uterine		Ovary		Total	
	Cases (CIR)	ASR	Cases (CIR)	ASR	Cases (CIR)	ASR	Cases (CIR)	ASR
2011	14 (0.55)	0.91	7 (0.27)	0.41	22 (0.86)	1.46	43 (1.69)	2.78
2012	17 (0.67)	1.32	12 (0.47)	0.63	9 (0.35)	0.5	38 (1.49)	2.48
2013	20 (0.78)	1.47	15 (0.59)	1.25	6 (0.24)	0.51	41 (1.61)	3.22
2014	21(0.82)	1.59	10 (0.39)	0.74	10 (0.39)	0.75	41 (1.61)	3.07
2015	24 (0.94)	1.75	6 (0.24)	0.47	5 (0.20)	0.22	35 (1.37)	2.47
2016	6 (0.23)	0.43	9 (0.35)	0.63	10 (0.39)	0.58	25 (0.98)	1.87
2017	14 (0.55)	1.23	20 (0.78)	1.01	12 (0.47)	0.73	46 (1.80)	3.43
APC		0.43		5.64		−11.26		1.13
95 (CI)		−18.3, 23.5		−13.8, 29.4		−28.6,10.3		−7.4, 10.4
*P-value*		0.8	0.3	0.5				0.8
Total	116 (4.55)	8.7	79 (3.10)	5.14	74 (2.90)	6.75	269 (10.54)	19.32

Data extraction was based on the definition of the International Classification of Diseases, 10th edition (ICD-10): cervix (C53), uterus (C55), and ovary (C56).

Abbreviations. CIR: Crude incidence rate; ASIR: Age Standardized incidence rate; APC: Estimated annual percent change; CI: confidence interval.

*Age adjusted to the population pyramid net for 2014.

### ASR per 100 000 and APC of Cervical, Ovarian and Uterine cancer between 2011–2017

Table 2 shows the CIR and ASR for cervical, ovarian and uterine cancers in Eritrea. Desegregation of CIR and ASR per age grouping was also attempted. According to this analysis, the highest ASR for cervical cancer was reported in individuals between 50–59 years of age. Similarly, high ASR for uterine and ovarian cancers were reported in individuals >60 years of age.

**Table 2:** ASR rates per 100,000 and APCs of cervical, ovarian, and uterine cancer according to age group.

Gynaecologic Cancers	Age Group CIR (ASR)				
	<40	40–49	50–59	– 69	>70
**Overall**					
2011	0.80 (0.02)	4.55 (0.33)	6.82 (0.58)	7.65 (0.65)	9.21 (1.12)
2012	0.66 (0.02)	3.98 (0.29)	5.97 (0.50)	8.93 (0.76)	6.91 (0.84)
2013	0.23 (0.01)	7.40 (0.53)	9.38 (0.79)	8.93 (0.76)	11.51 (1.40)
2014	0.33 (0.01)	5.69 (0.41)	7.67 (0.65)	15.31 (1.30)	6.91 (0.84)
2015	0.33 (0.01)	6.83 (0.49)	6.82 (0.58)	8.93 (0.76)	2.30 (0.28)
2016	0.23 (0.01)	3.41 (0.25)	2.56 (0.22)	10.21 (0.87)	6.91 (0.84)
2017	0.51 (0.02)	2.85 (0.20)	7.67 (0.65)	20.41 (1.74)	11.51 (1.40)
Total (ASR)	0.1	2.5	3.97	6.84	6.72
**Cervix (C53)**					
2011	0.23 (0.15)	1.71 (0.22)	2.56 (0.25)	2.55 (0.17)	2.30 (0.12)
2012	0.14 (0.09)	2.85 (0.36)	4.26 (0.42)	1.28 (0.09)	6.91 (0.36)
2013	0.14 (0.09)	2.85 (0.36)	6.82 (0.68)	5.10 (0.34)	0.00 (0.00)
2014	0.14 (0.09)	2.85 (0.36)	5.12 (0.51)	7.65 (0.51)	2.30 (0.12)
2015	0.19 (0.12)	4.55 (0.58)	5.97 (0.59)	5.10 (0.34)	2.30 (0.12)
2016	0.05 (0.03)	1.14 (0.14)	0.00 (0.00)	3.83 (0.26)	0.00 (0.00)
2017	0.00 (0.00)	1.71 (0.22)	3.41 (0.34)	6.38 (0.43)	4.60 (0.24)
Total (ASR)	0.57	2.24	2.79	2.14	0.96
**Ovary (C56)**					
2011	0.42 (0.28)	1.71 (0.22)	3.41 (0.34)	3.83 (0.26)	6.91 (0.36)
2012	0.14 (0.09)	0.57 (0.07)	0.85 (0.08)	3.83 (0.26)	0.00 (0.00)
2013	0.00 (0.00)	1.71 (0.22)	0.85 (0.08)	1.28 (0.09)	2.30 (0.12)
2014	0.09 (0.06)	1.14 (0.14)	1.71 (0.17)	3.83 (0.26)	2.30 (0.12)
2015	0.09 (0.06)	0.57 (0.07)	0.00 (0.00)	1.28 (0.09)	0.00 (0.00)
2016	0.19 (0.12)	2.28 (0.29)	0.85 (0.08)	1.28 (0.09)	0.00 (0.00)
2017	0.28 (0.18)	0.00 (0.00)	1.71 (0.17)	3.83 (0.26)	2.30 (0.12)
Total (ASR)	0.79	1.01	0.92	1.31	0.72
**Uterus (C54−55)**					
2011	0.14 (0.00)	1.14 (0.08)	0.85 (0.07)	1.28 (0.11)	0.00 (0.00)
2012	0.33 (0.01)	0.57 (0.04)	0.85 (0.07)	3.83 (0.33)	0.00 (0.00)
2013	0.09 (0.00)	2.85 (0.20)	1.71 (0.14)	2.55 (0.22)	9.21 (1.12)
2014	0.09 (0.00)	1.71(0.12)	0.85 (0.07)	3.83 (0.33)	2.30 (0.28)
2015	0.00 (0.00)	1.71 (0.12)	0.85 (0.07)	2.55 (0.22)	0.00 (0.00)
2016	0.00 (0.00)	0.00 (0.00)	1.71 (0.14)	5.10 (0.43)	6.91 (0.84)
2017	0.23 (0.01)	1.14 (0.08)	2.56 (0.22)	10.21 (0.87)	4.60 (0.56)
Total (ASR)	0.02	0.64	0.78	2.51	2.8

Data extraction was based on the definition of the International Classification of Diseases, 10th edition (ICD-10): cervix (C53), uterus (C54−55), and ovary (C56).

Abbreviations. CIR: Crude incidence rate; ASIR: Age Standardized incidence rate; APC: Estimated annual percent change; CI: confidence interval.

*Age adjusted to the population pyramid net for 2014.

## Discussion

Registration of cancer cases is an incredibly difficult pursuit in Africa. This has undermined the public availability of solid data on cancer prevalence, incidence and mortality from the region. For a large proportion of countries in the region, even low-quality population-based cancer registries (PBCR) are unavailable. In addition, existing screening or diagnostic services are generally sporadic and are often undertaken by disconnected institutions. In Eritrea, the situation is slightly different. First, the country has a highly centralized health sector and the private sector participation is almost non-existent. As a consequence, data from a limited number of institutions (NHL for example) may adequately represent the prevailing situation. In our study, 883 biopsies of cervical, ovarian and uterine tumours were assessed at the pathology department from 2011–2017.

In the study, 269 cases of invasive tumours were reported. Benign tumours/entities were more frequent in younger individuals while the converse was true for older individuals (Fig. 1). The data also demonstrates that risk increases with age until approximately 60 to 69 years. The profile uncovered in this study mirrors the findings from a previous study where a near similar age – specific incidence profile for benign and malignant entities was reported^11^. The observed incidence curve is generally attributed to lifetime accumulation of carcinogenic exposures (e.g. excess body weight/or obesity, cigarette smoking, among others) and somatic mutations, as well as age-related changes to the immune system^12^.

The ASR for cervical cancer, the highest ASR was reported in individuals between 60–69 years of age. Interestingly, the latest GLOBOCAN 2018 report for Eritrea indicates that the ASR for Cervical cancer is 13.8 per 100 000^13^ – the rates were computed using sex-; site- and age-specific incidence data from neighboring countries^13^. Needless to say, the ASR for cervical cancer reported in this study is considerably low. Higher ASRs for cervical cancer (42.7 per 100, 000) has been reported in the region (Eastern Africa). However, recent estimates from GLOBOCAN reports have placed the figure at 27.6 per 100 000 for SSA; 16.4 per 100 000 for US and 11.9 per 100 000 for Asia^2^. Considerable country to country and/or within country (particularly rural-urban) variation has been observed for cervical cancer in SSA^14^. Reports from Ethiopia indicate that ASR for Cervix uteri for the period (2012–2013) was 18.2 per 100 000. In Nairobi, Kenya, and Eldoret, Kenya, the ASR were 43.3 per 100 000 (Period: 2007–2011) and 26.8 per 100 000 (Period: 2008–2011), respectively. In Uganda, the ASR for cervical cancer was 51.1 per 100 000 (Period: 2008–2012)^14^.

The large geographic variation in the global distribution of cervical cancer has been attributed to multiple factors. For instance, the high incidence of cervical cancer in low-HDI countries has been attributed to poor screening and treatment of precancerous lesions combined with high prevalence of high-risk/oncogenic Human Papilloma Virus (HPV) (HPV-16, 18 subtypes) and to a lesser extent HPV types 31, 33, 45, 52, and 58^15,16^. Other important cofactors include tobacco consumption, sexually transmitted diseases (STDs) or high-risk sexual behavior, poor sanitary conditions, high parity (>3 children), and immune-suppression due to malnutrition and/or Human Immunodeficiency Virus (HIV), among others^17,18^. On the other hand, the low incidence of cervical cancers in high HDI settings has partially been attributed to high HPV vaccination coverage; screening programs (cytology testing - [Papanicolaou procedure/Pap smear] and HPV-DNA testing) and aggressive treatment of pre-neoplastic cervical lesions^18^. However, we have to note that a high frequency of Pap test or VIA screenings is often not feasible due to economic, social, cultural reasons. As a result, the uptake of Pap smear screenings is often low in some regions. Irrespective, research indicates that countries where programmatic cytological screening has been implemented over the last decades, cervical cancer incidence and mortality rates have declined steadily. Moreover, records indicate that cervical cancer was the 4^th^ most common disease in women and the 7^th^ around the world, representing approximately 9 of every 10 deaths in low HDI settings where screening programs (including visual inspection with acetic acid [VIA]) are unavailable^4^.

Like most countries in Africa, PAP screening coverage in Eritrea is relatively low. Opportunistic and/or programmatic screening programs and HPV vaccines are largely unavailable (e.g. HPV-DNA testing, Colposcopic diagnosis and VIA are rare or nonexistent). Indeed, PAP smear was only recently introduced and the HPV vaccines are unavailable. In addition, treatment of pre-invasive lesions is marginal given the minimal utilization of screening services by women in the country. Therefore, the low ASR rates in this study cannot be attributed to a functional health care system and high population level awareness. In contrast, the possibility that our study and the GLOBOCAN report have underestimated the incidence of cervical in the country is hard to discount. This concern underscores the desperate need for research on cancer incidence and related risk factors in the country. Irrespective, a combination of public awareness of causal factors, early cancer detection by regular screening (VIA or PAP smears) with the incorporation of HPV DNA testing, and vaccination at reasonable costs will constitute key preventive tools against cervical cancer in the country.

The incidence of uterine and ovarian cancer was also evaluated. The data suggests that pooled incidence of ovarian and uterine cancer is 2.8 per 100 000 and 2.2 per 100 000, respectively. The frequencies are close to what has been reported for ovarian and uterine cancers in SSA–5.0 per 100 000 and 3.3 per 100 000, respectively^19^. However, higher ASR values for uterine and ovarian cancer have been reported by some investigators. Reports from Nairobi, Kenya indicate that ASR for ovarian and uterine cancer (Corpus uteri) was 6.6 per 100 000 and 6.0 per 100 000, respectively (Period: 2007–2011). In Uganda, the ASR for ovarian and uterine cancers were 7.2 per 100 000 and 5.3 per 100 000 (Period: 2008–2012), respectively^15^. In addition, analysis of trends did not demonstrate any significant change over the study duration. Individuals within the 60–69 years age group had the highest ASR for both ovarian and uterine cancers. The lowest ASR was observed in individuals <40 years. Analysis of trends did not demonstrate any significant change over the study duration. In general, the age-standardized frequencies observed in this study align with what has been reported by some investigators in the region^19^.

Overall, increased personal risk for both uterine (e.g. endometrial cancer) and ovarian cancers is linked to short and/or irregular menstrual cycles; menopausal hormone use (Estrogen); late age at menopause and polycystic ovarian syndrome (PCOS)^20,21^. In addition, high body mass index (BMI) is a well-established risk factor for certain types of endometrial cancers (Type I endometrial cancer) and ovarian cancers^22,23^. Indeed, an estimated 70% of uterine corpus cancers are attributable to insufficient physical activity, excess body weight and metabolic disease^23^. The contributory role of these risk factors in this population remains uncertain. The role of factors such as PCOS and Talcum powder use;^24,25^ cannot be established since the necessary information was not captured in the primary database. And although ovarian cancer has a high hereditary portion, the frequency of genetic risk factors such as hereditary non-polyposis colorectal cancer (HNPCC)/or Lynch syndrome, BRCA1 and BRCA2 mutant genes are unknown.

### Limitation of the study

This study has several limitations. First, the number of cases evaluated for specific cancers was small. This may have an impact on evaluation of trends. In addition, reliance on microscopically verified cases may lead to underestimation of cases. The incidence of cervical cancer may also be influenced by the low screening rates. In all, the true case load for cervical, ovarian and uterine cancers may higher. However, the study has several strengths. First, it uses data from a centralized repository that has been operational over a long duration of time. Data collection was also standardized and all the cases recorded in the data base were confirmed by a pathologist.

## Conclusion

Cancer is an increasing problem in Sub-Saharan Africa because of number of factors including aging, sun exposure, radiation exposure, chemicals and other substances, viruses, certain hormones, family history of cancer, alcohol, poor diet and being overweight can be mentioned. Despite this growing concern, information on incidence, mortality and associated risk factors is severely limited. This report provides important information about the incidence and trends of cervical, uterine and ovarian cancers in Eritrea. In sum, it’s our opinion that the ASR for cervical cancer in Eritrea is relatively low compared to the rates reported in the region. We also documented relatively low ASR for ovarian and uterine cancers. Irrespective, the possibility that the ASR estimates reported in this study have underestimated the true burden of cervical, ovarian and uterine cancer incidence in the country. As a consequence, there is an urgent need to develop a more comprehensive evidence base for cancer data. One of the best strategies for obtaining data is through the establishment of cancer surveillance systems in the form of high quality PBCR that systematically collect, collate, analyze, and report cancer data. For cervical cancer screening, VIA, PAP smear, and possibly, HPV DNA testing is recommended. Increasing access to HPV vaccines and aggressive treatment of precancerous lesions should also be instituted. Additional research on the complex interplay of social, environmental and genetic influences on cancer risk is also warranted.

## Method and Materials

### Study design and setting

We conducted a retrospective study by identifying all incident cases of cervical, Ovarian and Uterine cancers captured 2011–2017 in NHL pathology database (Polytech 8.37.C). At present, NHL serves as a referral facility for all hospitals in Eritrea and has the only histopathology and cytology laboratory in the country. Therefore, it can be presumed that all histopathological specimens from patients in Eritrea are processed at this facility. The information captured in the system includes demographic characteristics (sex and age); histopathology (morphology), primary site (topography), behavior, date of diagnosis, among others.

### Case definition and ascertainment

The primary sub sites were defined based on the International Classification of Diseases (ICD) for Oncology 3^rd^ edition (ICD-10)^26^. Based on this scheme, the 3 main sites were defined as follows: cervix uteri (C53), ovarian (C56) and uterus (C54–55). The International Classification of Diseases for Oncology, third edition (ICD-O-3) was used to select all malignant cases. The topography codes applied included the following. Topography Codes: Adenocarcinoma (ICDO-3: 8140–8144, 8480–8770, 8560) and Squamous cell carcinoma (ICD-O-3: 8051–8083, 8120]. For uterus, the following topography codes were employed: Squamous cell carcinoma (ICD-O-3: 8070); Adenocarcinoma (8140), Leiomyosarcoma (ICDO-3: 8890–8896), Adenosarcoma (ICDO-3: 8893), among others. Similarly, ovarian cancer categories were classified as follows: Adenocarcinoma (ICD-O-3: 8140, 8323), Squamous cell carcinoma (ICD-O-3: 8070), Clear cell carcinoma (ICD-O-3: 8310, 8323), Endometrioid carcinoma (ICD-O-3: 8380), Serous (ICD-O-3: 8441, 8442, 8460–61), Mucinous, among others. Data abstracted from the NHL pathology database was subsequently checked for consistency by verification (sex and ICD-O-3 morphology and topography) and consistency between items (age Vs incidence dates/years). In addition, benign neoplasms/or entities included the following: Cysts, leiomyomas, fibromas, fibrosis, hemangiomas, cystadenomas, polyps, myomas, teratomas (dermoid), carcinoma *in situ*, Brenner tumour benign, dysplasia, hyperplasia, among others.

### Completeness and data quality

Data completeness was evaluated using a number of strategies including stability of incidence rates over time. The use of mortality-to-incidence ratios was not possible due to lack of data on mortality. Evaluation of age-specific incidence curves for atypical fluctuations, such as precipitous drops in incidence in older age sub-groups was also undertaken. Representativeness of the data was also evaluated using residency.

### Data analysis

Anonymous data from NHL Polytech data base (Polytech 8.37.C) was used in this analysis. Incidence rates were age standardized to the world standard population and is expressed per 100,000 populations. Age-standardized rates (ASIRs) per 100,000 person-years were calculated using the direct method and the world standard population^27^. In this analysis, 10-year age bands (<40, 40–49, 50–59, 60–69, 70–79, >80 years) was employed. Annual percentage change (APC) in rates were quantified using the National Cancer Institute’s Surveillance, Epidemiology, and End Results (SEER) Joinpoint Regression Program (V.4.5.0.1, Statistical Methodology and Applications Branch, Surveillance Research Program, National Cancer Institute, Bethesda, MD, USA)^28^. Accordingly, APC in rates between trend - change points (e.g. Joinpoint segment) was calculated using the following formula: exp(β)−1] × 100. The regression coefficient (β) was estimated from a linear regression between logarithmic-transformed age-standardized cancer rates per calendar year. The Monte Carlo permutation method was used for tests of significance. The 95% confidence intervals (CIs) were obtained with standard error from the fit of the regression and the t-distribution function. All p-values for significance testing of APC = 0 were two-sided, and considered to be of statistical significance when p < 0.05.

### Ethical approval and consent

Ethical clearance was obtained from the Health Research Ethics and Protocol Review Committee of the Ministry of Health and informed consent was waived by the committee therefore the records at the pathology department were made anonymous via de-identification.

## Supplementary information

Dataset 1.

## Data Availability

All data generated or analyzed during this study are included in this published article (and its Supplementary information files).

## References

[1] Global Burden of Disease Cancer Collaboration, “The Global Burden of Cancer. JAMA Oncol. 2015, 1 (4): 505–507 (2015). 10.1001/jamaoncol.2015.0735 PMC4500822 26181261

[2] International Agency for Research on Cancer. GLOBOCAN 2012: estimated cancer incidence, mortality and prevalence worldwide in 2012. Available from: http://globocan.iarc.fr/Default.aspx. (2012).

[3] Jemal A (2011). Global cancer statistics. CA Cancer J. Clin..

[4] Ferley, J. *et al*. GLOBOCAN 2008 v1.2, Cancer Incidence and Mortality Worldwide: IARC Cancer Base No. 10 [Internet]. Lyon, France: International Agency for Research on Cancer; Available from: http://globocan.iarc.fr (2010).

[5] Rahman R (2019). Cervical cancer screening decentralized policy adaptations: an African – Rural Context –Specific Systematic Literature review. Glob. Health Action..

[6] Bray F (2018). Global Cancer Statistics 2018: GLOBOCAN Estimates of Incidence and Mortality Worldwide for 36 Cancers in 185 Countries. Ca Cancer J. Clin..

[7] Goff B (2007). Development of an ovarian cancer symptoms index. Possibilities early detection. Cancer..

[8] Vaccarella S, Laversanne M, Ferlay J, Bray F (2017). Cervical cancer in Africa, Latin America and the Caribbean, and Asia: Regional inequalities and changing trends. Int. J. Cancer.

[9] Ferlay J, *et al*. Cancer incidence and mortality worldwide: Sources, methods and major patterns in GLOBOCAN 2012. International Journal of Cancer. **136**, E359–E86. 10.1002/ijc.29210.PMID:25220842 (2015). 10.1002/ijc.29210 25220842

[10] Louie, K. S., de Sanjose, S. & Mayaud, P. Epidemiology and prevention of human papillomavirus and cervical cancer in sub-Saharan Africa: a comprehensive review. Trop Med Int Health. 14(10):1287–302. 10.1111/j.1365-3156.2009.02372.x PMID:19772550 (2009). 10.1111/j.1365-3156.2009.02372.x 19772550

[11] Oh C-M (2013). Trends in the Incidence of *In Situ* and Invasive Cervical Cancer by Age Group and Histological Type in Korea from 1993 to 2009. Plos one.

[12] DeSantis CE (2019). Cancer Statistics for Adults Aged 85 Years and Older. Ca Cancer J. Clin. 2019.

[13] GLOBOCAN. Cancer Fact Sheets: prostate cancer. Available online: http://globocan.iarc.fr/old/FactSheets/cancers/prostate-new.asp (2017).

[14] Parkin, D. M. *et al*. Cancer in Sub-Saharan Africa. IARC Scientific Publications. No. 167 (2018).

[15] Bray F (2005). Incidence trends of adenocarcinoma of the cervix in 13 European countries. Cancer Epidemiol. Biomarkers Prev..

[16] Crosbie EJ (2013). Human papillomavirus and cervical cancer. Lancet..

[17] Vaccarella S, Lortet-Tieulent J, Plummer M, Franceschi S, Bray F (2013). Worldwide trends in cervical cancer incidence: impact of screening against changes in disease risk factors. Eur. J. Cancer..

[18] Shetty MK, Langatto-Filho A (2011). Early Detection of Breast, Cervical, Ovarian and Endometrial Cancers in Low Resource Countries: An Integrated Approach. Indian. J. Surg. Oncol..

[19] Tessema S. M. *et al*. Estimates of Cancer Incidence in Ethiopia in 2015 Using Population-Based. Journal of Global Oncology. 10.1200/JGO.17.00175 (2018). 10.1200/JGO.17.00175 PMC6223441 30241262

[20] Cramer Daniel W. (2012). The Epidemiology of Endometrial and Ovarian Cancer. Hematology/Oncology Clinics of North America.

[21] Sanch-Garnier H (2013). “Overview of cervical cancer screening practices in the extended middle east and North Africa Countries,”. Vaccine..

[22] Weiderpass E (2000). Body size in different periods of life, diabetes mellitus, hypertension, and risk of postmenopausal endometrial cancer (Sweden). Cancer Causes Control..

[23] Cramer DW (2012). The Epidemiology of Endometrial and Ovarian Cancer. Hematol. Oncol. Clin. North. Am..

[24] Cramer DW (1999). Genital talc exposure and risk of ovarian cancer. Int. J. Cancer..

[25] Vaccarella S, Laversanne M, Ferlay J, Bray F (2017). Cervical cancer in Africa, Latin America and the Caribbean, and Asia: Regional inequalities and changing trends. Int. J. Cancer.

[26] World Health Organization. International classification of diseases for oncology, 3rd ed (ICD-O-3). Available from: http://codes.iarc.fr/codegroup/1. (2000).

[27] World Health Organization (Age Standardization of Rates) https://www.who.int/healthinfo/paper31.pdf (Accessed 1 Mar 2020).

[28] Joinpoint Regression Program; version 4.5.0.1; Statistical Methodology and Applications Branch, Surveillance Research Program; National Cancer Institute: Bethesda, MD, USA. Available: file:///C:/Users/u1104044/Downloads/ Joinpoint_ Help_ 4. 5. 0. 1(1). (2017).

